# Gait improvement with wearable cyborg HAL trunk unit for parkinsonian patients: five case reports

**DOI:** 10.1038/s41598-023-33847-z

**Published:** 2023-04-28

**Authors:** Akira Uehara, Hiroaki Kawamoto, Hisamasa Imai, Makoto Shirai, Masatomi Sone, Sachiko Noda, Shigeto Sato, Nobutaka Hattori, Yoshiyuki Sankai

**Affiliations:** 1 Faculty of Engineering, Information and Systems, University of Tsukuba, Ibaraki, 305-8573 Japan; 2 Department of Neurology, Tokyo Rinkai Hospital, Tokyo, 134-0086 Japan; 3 Department of Rehabilitation, Limited Company Jin, Saitama, 341-0003 Japan; 4 Unaffiliated, Saitama, 359-0025 Japan; 5 Department of Neurology, Juntendo University Graduate School of Medicine, Tokyo, 113-8431 Japan; 6 Center for Cybernics Research, University of Tsukuba, Ibaraki, 305-8573 Japan

**Keywords:** Parkinson's disease, Mechanical engineering

## Abstract

Cybernic treatment involves the generation of an interactive bio-feedback loop between an individual’s nervous system and the worn cyborg Hybrid Assistive Limb (HAL); this treatment has been applied for several intractable neuromuscular disorders. Thus, it is of interest to determine its potential for parkinsonian patients. This study confirmed the feasibility of using a HAL trunk unit to improve parkinsonian gait disturbance. HAL establishes functional and physical synchronization with the wearer by providing lateral cyclic forces to the chest in the form of somatosensory and motor cues. To confirm the feasibility of its use for improving parkinsonian gait disturbances, we conducted experiments with three Parkinson’s disease patients and two patients with progressive supranuclear palsy. During the experiments, the immediate effect of the intervention was assessed; all participants exhibited improvements in gait disturbance while wearing the HAL unit, and this improvement effect persisted without the HAL unit in two participants. Afterward, based on the assessment, we conducted a continuous intervention for one participant. In this intervention, the number of steps in the final experiment was significantly decreased compared with the initial state. These findings suggest that the proposed method is an option for treating parkinsonian patients to generate somatosensory and motor cues.

## Introduction

Parkinsonian gait disturbances decrease mobility and cause fear of falls. A staggering gait leads the body to wobble from side to side. A small stepping gait is a phenomenon in which the heel of one foot does not extend beyond the toe of the other foot while walking, and the stride length of each step is less than a foot in length. A festinating gait involves gradual forward momentum that makes it difficult to stand still. Freezing of gait is characterized by a lack of or marked decrease in the forward progression of the lower limbs that intermittently occurs for a short period despite the will to walk; staggering gait is the initial symptom. Staggering gait and festinating gait frequently induce freezing of gait^1^. These gait disturbances lead to falls^2^. The symptoms are considered to be primarily due to postural dysfunction (bending forward of the trunk), impaired rhythm formation (difficulty in continuing periodic movements), akinesia (decrease in movement amplitude), and a sequence effect (gradual decrease in stride length) caused by different network failures associated with the basal ganglia^3^. However, the relationship between gait disturbances and the physiology of these various functional declines remains unclear^1–6^. The symptom progression and physical characteristics significantly differ between patients; symptoms exhibit intraday or daily variations and are markedly influenced by the surrounding environment and the associated psychological states. Various approaches have been proposed to treat the motor symptoms of Parkinson's disease. In general, drug therapy with levodopa (l-dopa) and surgical treatment with deep brain stimulation are selected based on various factors, including the progression of symptoms, treatment efficacy, side effects, and patient satisfaction^3,7–9^.

External cues that promote increased stride length and gait maintenance through visual, auditory, and superficial sensory stimulation provide immediate improvement; these cues are most widely used in parallel with drug and medical treatment^2^. Instructions for straddling movements with external cues such as tape on the floor, rhythmic vocal cues, and tapping on the buttocks are used daily. However, these cues do not provide physical force due to the functional declines in postural control and rhythm generation that cause gait disturbances and because the motor commands for voluntary control related to the gait are generated by unconventional processes that circumvent damaged areas^10,11^. Moreover, parkinsonian patients have difficulty shifting their center of gravity (COG) in a lateral direction at the start of gait and difficulty turning due to a functional decline in the central nervous system (CNS) involved in movement generation and coordination^12,13^. Therefore, visual, auditory, and superficial sensory cues have not been established as a standardized treatment method, and their effectiveness varies among patients. However, these cues have been reported to be effective^11^. Physical therapists may hold the patient under the arms to help them walk, keeping the trunk upright and swaying the patient rhythmically from side to side. The intervention compensates postural control and rhythm generation with somatosensory and motor cues, synchronizing the patient’s motions and cues. This approach promotes rhythmical and lateral movement of the center of mass (COM) and is recommended for parkinsonian patients because it prevents FOG^14,15^. For these reasons, somatosensory and motor cues (i.e., whole-body movements with force synchronized with the patient’s intention) rather than visual, auditory, and superficial stimulation are preferable for improving gait disturbances by shifting the COG. Previous joint assist devices have provided cues to induce forward steps in patients^16,17^, although these devices have used sagittal and noncyclic force rather than the recommended lateral cyclic force. Therefore, a wearable system that provides lateral cyclic force to the user may effectively improve gait and movement similar to the somatosensory and motor cues provided by physical therapists. For such interventions to work, the system’s assistance and the user’s motion need to be synchronized, similar to the cues of physical therapists.

The wearable cyborg Hybrid Assistive Limb (HAL) functions in accordance with the motor intention of the wearer and ideal movement patterns as if it were a part of the body by seamless adjustment between voluntary and autonomous movement. The process that realizes the fusion of a wearer and HAL via the bidirectional exchange of neural information, such as motor unit potentials, and dynamic mechanical information, such as coordinates between the wearer’s nervous system and HAL, involves interactive bio-feedback (iBF)^18^. When efferent and afferent signals are synchronized with proper timing, such as during movement in which humans swing their legs and control their posture based on vision, balance, and proprioception, the neural network with high activity is selected based on the theory of neural group selection because the stimuli sensed by all receptors are repeated at the appropriate time^19^. Additionally, repetition enhances the transmission efficiency of synapses based on the Hebbian rule^20^. Previous studies of HAL have shown that cybernic treatments based on iBF effectively improve the gait function of patients with several intractable neurological and neuromuscular disorders^21–23^. For these reasons, HAL (which provides lateral and cyclic forces based on conventional findings regarding physical therapy) generates somatosensory and motor cues and has the potential to enhance iBF because HAL amplifies the lateral sway of patients, allowing them to achieve near-normal voluntary movement. Previously developed devices that affect small muscle groups are capable of promoting plasticity and CNS map modification^24^; however, proposed interventions related to gait and postural control during whole-body movement can be effectively promoted by recommended somatosensory and motor cues^14,25–27^. Although wearable cyborgs and robots have been applied in trials to improve the gait of patients with Parkinson’s disease^28,29^, it is challenging for these methods to directly assist postural control and rhythm generation in the frontal plane, as recommended by physical therapists. Considering that the weight and inertia of the upper/lower limb exoskeleton can inhibit remaining motor functions, it is critical to develop new methods in parallel with conventional approaches. In this study, we confirmed the feasibility of using the wearable cyborg HAL trunk unit, which promotes lateral sway synchronization with a wearer’s motion, to improve the gait disturbance of parkinsonian patients.

## Methods

### Participants

We conducted gait experiments with five parkinsonian patients to confirm the feasibility of using HAL to improve gait disturbance. Table 1 presents the participants’ information; the four gait disturbances caused by disease type are listed. Four participants could walk independently; the participant in Case 5 could not due to severe trunk dystonia. Antiparkinson drugs and dosages were as follows: Case 1 took levodopa (700 mg-carbidopa 70 mg/day), rotigotine (27 mg (patch)/day), and selegilline hydrochloride (7.5 mg/day). Case 3 took levodopa (450 mg-benserazide 112.5 mg/day), trihexyphenidyl hydrochloride (3 md/day), amantadine hydrochloride (150 mg/day), and zonisamide (100 mg/day). Case 4 was treated with levodopa (600 mg-carbidopa 60 mg/day) and clonazepam (0.5 mg/day).

**Table 1:** Summary of participant physical and clinical characteristics.

Case	Age	Sex	Weight	Disease	DI (years)	HY	UPDRS	AE	Drug treatment
1	69	F	46 kg	PD	7	2.5	2/4	–	IS, LC, RO, SH,
2	72	M	64 kg	PSP	1	3	3	Cane	-
3	65	M	70 kg	PD	42	3	1	–	AH, DR, LB, TH, ZNS
4	80	M	72 kg	PD	6	4	2	Cane	CZP, LC
5	65	M	75 kg	PSP	5	5	4	EW	–

*AE* usage of assistive equipment in daily life, *AH* amantadine hydrochloride, *CZP* clonazepam, *DI* duration of illness, *DR* droxidopa, *EW* electric wheelchair, *F* female, *FG* festinating gait, *FOG* freezing of gait, *GD* gait disturbance, *HY* Modified Hoehn and Yahr Scale, *IS* istradefylline, *LB* levodopa-benserazide, *LC* levodopa-carbidopa, *M* male, *PD* Parkinson’s disease, *PSP* progressive supranuclear palsy–pure akinesia with gait freezing, *RO* rotigotine, *SG* staggering gait, *SH* selegiline hydrochloride, *SSG* small stepping gait, *TH* trihexyphenidyl hydrochloride, *UPDRS* Unified Parkinson’s Disease Rating Scale, item 14 (freezing of gait), *ZNS* zonisamide.

### The wearable cyborg hybrid assistive limb (HAL) trunk unit

**Figure 1:**
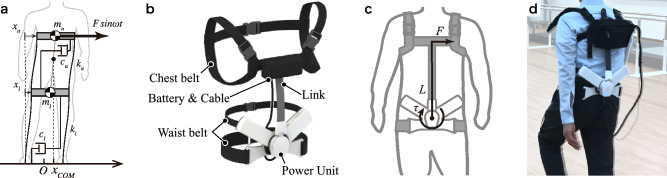
A wearable cyborg Hybrid Assistive Limb (HAL) trunk unit can provide lateral cyclic force to the chest. (**a**) Simplified model of the frontal plane. (**b**) Outside of the HAL unit. (**c**) Force transmission by HAL. (**d**) Walking with the HAL unit. The rendering function of SOLIDWORKS 2018 was used to create part of the image. We previously developed a system and evaluated the basic function of parkinsonian patients as a case study^30,31^.

Figure 1 shows the wearable cyborg HAL trunk unit, and the parameters of lateral cyclic force are shown in Table 2. HAL started providing lateral cyclic force to the wearer’s chest during a standing posture and continued assistance during walking, thereby supporting rhythm generation and COM shifting based on the recommended approach in physical therapy. HAL’s force led to lateral bending of the upper body, and whole-body movement in the frontal plane was promoted by the abduction and ankle joints. In this study, the amplitude and period of lateral cyclic force were derived from the wearer’s body weight and gait cycle based on a simple analytical model of the physical and physiological features of parkinsonian patients and refined by interviewing the physical therapist and each participant. HAL’s force and the wearer’s motion were synchronized because of resonance of somatosensory and motor cues at a natural frequency (i.e., the wearer’s normal gait cycle). The relationship between the participant’s features and the force parameters is expressed as

$$\frac{M}{5}\left[\begin{array}{cc}3& 0\\ 0& 2\end{array}\right]\left[\begin{array}{c}\ddot{{x}_{u}}\\ \ddot{{x}_{l}}\end{array}\right]+\left[\begin{array}{cc}{c}_{u}& -{c}_{u}\\ -{c}_{u}& {c}_{u}+{c}_{l}\end{array}\right]\left[\begin{array}{c}\dot{{x}_{u}}\\ \dot{{x}_{l}}\end{array}\right]+\left[\begin{array}{cc}{k}_{u}& -{k}_{u}\\ -{k}_{u}& {k}_{u}+{k}_{l}\end{array}\right]\left[\begin{array}{c}{x}_{u}\\ {x}_{l}\end{array}\right]=\left[\begin{array}{c}F\\ 0\end{array}\right],$$

$${\left[\begin{array}{cc}{k}_{u}& {c}_{u}\end{array} \begin{array}{cc}{k}_{l}& {c}_{l}\end{array}\right]}^{T}={\left[\begin{array}{cc}\frac{3M{{\omega }_{0}}^{2}}{5}& \frac{6M{\omega }_{0}}{5}\sqrt{1-{\left(\frac{\omega }{{\omega }_{0}}\right)}^{2}}\end{array} \begin{array}{cc}\frac{2M{{\omega }_{0}}^{2}}{5}& \frac{4M{\omega }_{0}}{5}\sqrt{1-{\left(\frac{\omega }{{\omega }_{0}}\right)}^{2}}\end{array}\right]}^{T},$$

where $M$ is the body weight; ${x}_{u}$ is the position of the upper body’s COM; ${x}_{l}$ is the position of the lower body’s COM; ${c}_{u}$ is the coefficient of viscosity of the upper body; ${k}_{u}$ is the coefficient of elasticity of the upper body; ${c}_{l}$ is the coefficient of viscosity of the lower body; ${k}_{l}$ is the coefficient of elasticity of the lower body; ${\omega }_{0}$ is the angular frequency of gait without disturbance; $\omega$ is the angular frequency of the cyclic assist; and $F$ is the amplitude of the lateral cyclic force.

**Table 2:** Parameters of the lateral cyclic force.

Case	Amplitude (N)	Frequency (s)
1	5.2	1.1
2	8.5	1.2
3	10.1	1.21
4	4.3	1.5
5	5.0	1.1

To provide the necessary lateral force to the wearer’s upper body to affect the COG (located near the wearer’s chest), HAL was attached by belts at the chest and waist; a power unit fixed at the waist transmitted forces to the chest via a link. The rotation axis of the power unit was perpendicular to the wearer’s back, and the power unit was cyclically rotated forward and backward to provide cyclic force to the chest belt. The length of the waist belt, chest belt, and link can be adjusted according to the wearer’s body size. The length and position of the belt were adjusted such that the lumbar belt was on the superior anterior iliac spine and the tip of the link was on the fifth thoracic vertebra. By adjusting the length of the belt and links, the wearer’s trunk extension was facilitated, similar to physical therapy. The lateral cyclic force was smoothed to prevent balance disruption due to the rapid increase in the provided force. The output torque is expressed as

$$\tau =\left\{\begin{array}{c}\frac{\omega }{\pi }tLF\mathrm{sin}\omega t\\ LF\mathrm{sin}\omega t\end{array}\right. \begin{array}{c}\left(0\le t<\frac{\pi }{\omega }\right)\\ \left(t\ge \frac{\pi }{\omega }\right)\end{array},$$

where $\tau$ is the torque provided by the power unit of HAL and $L$ is the length of the link.

These parameters were calculated according to the participants’ body weight and gait cycle based on a simple analytical model of parkinsonian patients' physical and physiological features.

### Measurements

This study involved three trials in one day: Trial 1 (preintervention without HAL); Trial 2 (during intervention with HAL); and Trial 3 (postintervention without HAL). Each trial was performed three times, and the interval between each trial was approximately fifteen minutes. Moreover, the experiment was conducted three times over five months after the first experiment day to confirm the feasibility of using repeated HAL intervention to achieve persistent gait improvements for progressive symptoms. The criteria for the continuous intervention were as follows: (1) on the first day, patients exhibited improvement in Trial 3 compared with Trial 1, and (2) the participant, physical therapist, and medical doctor agreed to participate in the long-term intervention. In the continuous intervention, the intervals between Day 1 and Day 2 and between Day 3 and Day 4 were one month, and the interval between Day 2 and Day 3 was four months.

**Figure 2:**
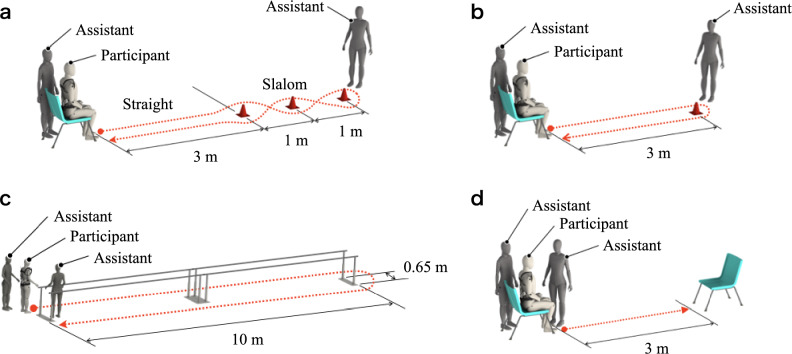
Experimental environments. (**a**) Slalom test, used to measure staggering behavior. (**b**) TUG test, used to measure mobility and balance. (**c**) U-shape test, used to measure festinating behavior. (**d**) The straight test, a simplified condition based on the TUG test. TUG, timed up and go.

Table 3 and Fig. 2 show the evaluation indices and experimental environments, which varied according to the symptoms of each participant. In this study, symptoms and types of gait disturbances differed among participants; additionally, fatigue can cause gait disturbance. For these reasons, the evaluation indices and experimental environment for each case were set before the experiment to verify the repeatability of gait improvement while minimizing the physical and mental burdens. To measure the evaluation index for each gait disturbance, we constructed environments based on the 10-m walking test and timed-up-and-go test, which are generally used for clinical evaluations. The center of foot pressure was measured with a frequency of 100 Hz using the Pedar-X pressure distribution measurement system (novel GmbH, Munich, Germany) to measure the center of pressure (COP). Symptoms of Parkinson’s disease begin on one side, gradually become more severe, and finally appear bilaterally. For this reason, Cases 1, 3, and 4 were evaluated using data from the affected side (diagnosed by a medical doctor), and Case 5 was evaluated using data from both sides. The timing of heel contact, determined by video recordings, was used to measure the gait cycle and the number of steps. To understand the pathological condition and reduce anxiety about participation in the experiment, the experimental environment was confirmed before the day of Trial 1. If the HAL unit operated unexpectedly due to a malfunction or other defect, the assistant halted the experiment and turned off the HAL unit. The assistant also monitored the participants at all times during the experiment and held the participants to prevent falling if the participants lost balance, thereby maximizing safety considerations without influencing lateral swing. The assistants placed the HAL unit on the participants to ensure proper fit and effective operation. However, HAL was designed to be donned independently by the user. In Cases 1, 3, and 4, we conducted an experiment under the positive effect of dopa.

**Table 3:** Participant evaluation indices and experimental environments.

Case	Evaluation index	Environment
1	Maximum value of lateral COP (right side only)	Slalom
2	Number of steps	TUG test
3	Linear trendline slope of gait cycle	U-shape
4	Time difference between maximum and minimum gait cycle	TUG test
5	Maximum value of lateral COP (right and left side)	Straight

*COP* center of pressure, *TUG* timed up and go.

### Data analyses

In this study, the results are not shown as group aggregates but rather for each participant because each participant had a different gait disturbance, and the evaluation indices could not be standardized. All data are expressed as the mean and standard deviation (SD). To validate HAL-induced gait improvement, comparisons of gait data between preintervention (i.e., Trial 1 on Day 1, the baseline) and other conditions were made using Dunnett’s multiple comparisons tests. Significance was considered in comparisons with P < 0.05. R 3.6.0 (The R Foundation, Vienna, Austria) was used for data extraction and statistical analyses.

### Ethical approval

The study was conducted with the approval of the Research Ethics Committee of the Graduate School of Systems and Information Engineering, University of Tsukuba, and informed consent was obtained from the participants. All experiments were conducted according to the tenets of the Declaration of Helsinki.

## Results

### Case 1: PD with staggering gait (modified Hoehn and Yahr Scale: 2.5)

**Figure 3:**
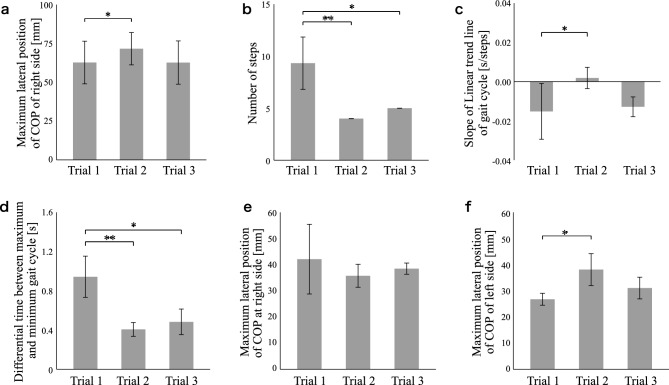
Experimental results for each participant. Each experiment consisted of preintervention without HAL (Trial 1), intervention with HAL (Trial 2), and postintervention without HAL (Trial 3). The bar graphs indicate the average of each index, and error bars indicate the standard deviation of the value. **P < 0.01, *P < 0.05; absence of an asterisk indicates P > 0.05. (**a**) The maximum lateral position of the COP on the right side in the slalom area for Case 1. (**b**) Number of steps in the turning area for Case 2. (**c**) Slope of the linear trendline of the gait cycle in the out and back areas for Case 3. (**d**) Time difference between the maximum and minimum gait cycles in the out, turn, and back areas for Case 4. (**e**) Maximum lateral position of the COP on the right side in the straight area for Case 5. (**f**) The maximum lateral position of the COP on the left side in the straight area for Case 5. *COP* center of pressure; *HAL* hybrid assistive limb.

Figure 3a shows the maximum lateral position of the COP on the right side during the slalom test. These values were 62.7 $\pm$ 13.4 mm, 71.6 $\pm$ 10.2 mm, and 62.7 $\pm$ 13.6 mm. There was a significant difference between Trial 1 and Trial 2. The results showed that the stability of the COM was improved due to increased COP movement while wearing the HAL unit.

### Case 2: PSP with small stepping gait (modified Hoehn and Yahr Scale: 3)

**Figure 4:**
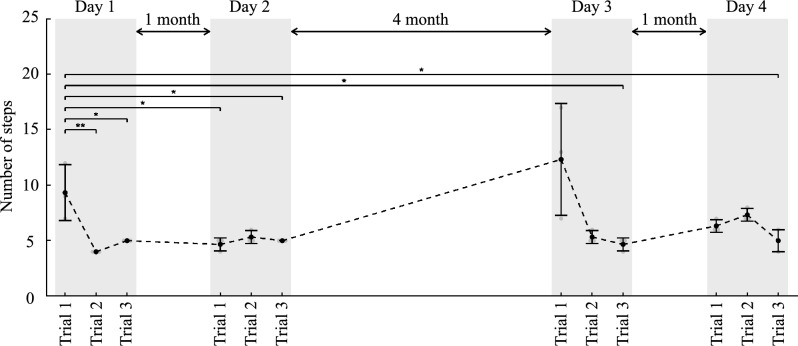
Experimental results of continuous intervention for Case 2 (progressive supranuclear palsy–pure akinesia with gait freezing). The black dots indicate the average number of steps in the turning area, and the error bars indicate the standard deviation of the value. **P < 0.01, *P < 0.05; absence of an asterisk indicates P > 0.05. Each trial consisted of preintervention without HAL (Trial 1), intervention with HAL (Trial 2), and postintervention without HAL (Trial 3).

Figure 3b shows a number of steps in the turning area. These values were 9.3 $\pm$ 2.1 steps, 4.0 steps, and 5.0 steps. There were significant differences between Trial 1 and Trial 2 and between Trials 1 and 3. The results showed that step lengths were increased, as indicated by the decreased number of steps while/after wearing the HAL unit. The participant was selected for the continuous intervention based on these assessments. Table 4 shows the participant’s physical features and HAL parameters in each trial. Figure 4 shows the number of steps in the turning area in the continuous intervention. On Day 2, these values were 4.7 $\pm$ 0.5 steps, 5.3 $\pm$ 0.5 steps, and 5.0 steps. On Day 3, these values were 12.3 $\pm$ 4.1 steps, 5.3 $\pm$ 0.5 steps, and 4.7 $\pm$ 0.5 steps. On Day 4, these values were 6.3 $\pm$ 0.5 steps, 7.3 $\pm$ 0.5 steps, and 5.0 $\pm$ 0.8 steps. There were significant differences between Trial 1 on Day 1 and Trial 1 on Day 2, Trial 3 on Day 2, Trial 3 on Day 3, and Trial 3 on Day 4. The results showed that the effects persisted when not wearing the HAL unit.

**Table 4:** Parameters of continuous intervention in Case 2.

Day	Body weight (kg)	Gait cycle (s)	Amplitude (N)	Frequency (s)
1	64	1.1	7.6	1.21
2	64	1.05	7.6	1.21
3	64	1.0	5.2	1.15
4	64	1.0	7.8	1.15

### Case 3: PD with festinating gait (modified Hoehn and Yahr Scale: 3)

Figure 3c shows the slope of the linear trendline of the gait cycle in the out and back areas. These values were − 0.02 $\pm$ 0.01 s/steps, 0.002 $\pm$ 0.005 s/steps, and − 0.01 $\pm$ 0.005 s/steps. There was a significant difference between Trial 1 and Trial 2. The results showed that festination was reduced while wearing the HAL unit, as indicated by the increasing slope of the linear trendline of the gait cycle.

### Case 4: PD with freezing of gait (modified Hoehn and Yahr Scale: 4)

Figure 3d shows the time differences between the maximum and minimum gait cycles in the out, turn, and back areas. These values were 0.94 $\pm$ 0.17 s, 0.41 $\pm$ 0.057 s, and 0.48 $\pm$ 0.11 s. There were significant differences between Trial 1 and Trial 2 and between Trial 1 and Trial 3. The results showed that the stability of rhythmic steps increased because of decreased gait cycle variability while/after wearing the HAL unit. The participant was not selected for the continuous intervention based on the assessments by the participant, physical therapist, and medical doctor.

### Case 5: PSP with freezing of gait (modified Hoehn and Yahr Scale: 5)

Figure 3e,f show the maximum lateral position of the COP on the right and left sides in the straight area. The right-side values were 42.1 $\pm$ 10.9 mm, 35.8 $\pm$ 3.6 mm, and 38.5 $\pm$ 1.7 mm. The left-side values were 26.8 $\pm$ 1.8 mm, 38.1 $\pm$ 5.0 mm, and 31.1 $\pm$ 3.4 mm. There was a significant difference in left-side values between Trial 1 and Trial 2. The results showed that the stability of the COM on the left side was improved due to the increased COP shifting on the left side while wearing the HAL unit. In this case, the participant required forward assistance from a physical therapist while using the HAL because of severe trunk dystonia. The support by the physical therapist was not related to rhythm because the assistant support involved static holding rather than lateral cyclic force.

## Discussion

Given the significant differences between Trial 1 and Trial 2, intervention with HAL was effective in improving the index associated with each gait disturbance. The significant differences between Trial 1 and Trial 3 in Case 2 and Case 4 indicated that gait improvement persisted without the HAL unit. In particular, the continuous intervention (in Case 2) led to a decreased number of steps on Day 1, which was maintained until Trial 1 of Day 2. On Day 4, as well as Day 3, the number of steps in Trial 3 decreased significantly, although no effect of antiparkinsonian drugs was observed. No safety issues with the use of HAL occurred. Therefore, we confirmed the feasibility of using HAL to improve parkinsonian gait disturbances. HAL is an advantageous new approach that can improve progressive gait disturbances in parkinsonian patients, who generally have restrained gait independence or are injured by falls due to these diseases. Moreover, this method can be applied to a wide range of assistive devices that provide physical assistance for whole body movement^32^.

The proposed method in this study provides cyclical intervention during whole-body movement via somatosensory and motor cues without interfering with the wearer’s residual gait function to achieve voluntary gait, thereby increasing whole-body movement (including COM shifting based on motion intention), which was inhibited by ataxia. Therefore, the efferent and afferent signals were amplified repeatedly and synchronously utilizing the basic principal of rhythmic physical cues to construct an iBF loop in which “motor command signals reduced by CNS dysfunction (i.e., efferent signals)” and “intrinsic sensory information obtained from numerous muscle groups in the trunk and postural control system and vestibular and visual sensory information caused by whole-body movement (i.e., afferent signals)” achieved one-to-one correspondence. Additionally, the results of the participant in Case 4 indicate that the returning sensory feedback to the thalamus was amplified by whole-body movement, and the projection to the cerebral cortex and subthalamic nucleus was optimized^33–35^, which may have resulted in smoother movements postintervention without the HAL unit. Thus, the method established an iBF loop utilizing somatosensory and motor cues by not only providing sensory information (such as auditory and superficial sensation, including pressure and touch) but also enhancing afferent signals, which are difficult to alter with conventional devices, and improving the plasticity of the CNS, which is involved in posture adjustment and motor command and leads to parkinsonian gait disturbances. To verify the detailed mechanisms and effects of the proposed method for use in evidence-based medicine, brain imaging is needed to determine the causal relationship between the proposed method and CNS plasticity; in addition, statistical verification from long-term intervention trials and randomized controlled trials including large numbers of parkinsonian patients is needed.

In this study, the gait disturbances of three participants were improved only while wearing the HAL unit. Further enhancement of the quality of life in such patients by daily use of HAL intervention may be achieved after determination of configurations such as the weights of the actuator and battery and the feasibility of operating and wearing the HAL unit during daily activities and situations. Additionally, the modulation of parameters regarding lateral cyclic force is essential because a wide range of diseases cause parkinsonian gait disturbances, and the symptom progression, physical characteristics, and intraday/daily occurrences vary among patients. The amplitude and frequency of lateral cyclic force application of the developed HAL unit are calculated according to symmetrical and static parameters on the left and right sides based on predefined wearer information. For this reason, the HAL unit cannot flexibly respond to changes in a patient’s gait due to fatigue, decreased efficacy of medications over time, or changes in physical characteristics. HAL dynamically fits parameters related to lateral cyclic force according to the wearer’s physical and biological conditions, such as bioelectrical signals, gait cycle, and ground reaction force, to achieve a comfortable and optimal intervention.

## Data Availability

The datasets generated and/or analyzed during the current study are not publicly available due to privacy and ethical restrictions but are available from the corresponding author on reasonable request.
