# Author Correction: Gait improvement with wearable cyborg HAL trunk unit for parkinsonian patients: five case reports

**DOI:** 10.1038/s41598-023-35421-z

**Published:** 2023-05-22

**Authors:** Akira Uehara, Hiroaki Kawamoto, Hisamasa Imai, Makoto Shirai, Masatomi Sone, Sachiko Noda, Shigeto Sato, Nobutaka Hattori, Yoshiyuki Sankai

**Affiliations:** 1 Faculty of Engineering, Information and Systems, University of Tsukuba, Ibaraki, 305‑8573 Japan; 2 Department of Neurology, Tokyo Rinkai Hospital, Tokyo, 134‑0086 Japan; 3 Department of Rehabilitation, Limited Company Jin, Saitama, 341‑0003 Japan; 4 Unaffiliated, Saitama, 359‑0025 Japan; 5 Department of Neurology, Juntendo University Graduate School of Medicine, Tokyo, 113‑8431 Japan; 6 Center for Cybernics Research, University of Tsukuba, Ibaraki, 305‑8573 Japan

Correction to: *Scientific Reports* 10.1038/s41598-023-33847-z, published online 28 April 2023

The original version of this Article contained an error in the Introduction, where reference 14 was cited incorrectly.

“Previous studies of HAL have shown that cybernic treatments based on iBF effectively improve the gait function of patients with several intractable neurological and neuromuscular disorders^14,21,22,23^.”

now reads:

“Previous studies of HAL have shown that cybernic treatments based on iBF effectively improve the gait function of patients with several intractable neurological and neuromuscular disorders^21,22,23^.”

The original Article has been corrected.

